# Short-Term Proteasome Inhibition: Assessment of the Effects of Carfilzomib and Bortezomib on Cardiac Function, Arterial Stiffness, and Vascular Reactivity

**DOI:** 10.3390/biology13100844

**Published:** 2024-10-21

**Authors:** Callan D. Wesley, Annarita Sansonetti, Cedric H. G. Neutel, Dustin N. Krüger, Guido R. Y. De Meyer, Wim Martinet, Pieter-Jan Guns

**Affiliations:** Laboratory of Physiopharmacology, Faculty of Medicine and Health Sciences, Faculty of Pharmaceutical, Biomedical and Veterinary Sciences University of Antwerp, Campus Drie Eiken, 2610 Antwerp, Belgium; annarita.sansonetti@studenti.unipd.it (A.S.); cedric.neutel@uantwerpen.be (C.H.G.N.); dustin.kruger@uantwerpen.be (D.N.K.); guido.demeyer@uantwerpen.be (G.R.Y.D.M.); wim.martinet@uantwerpen.be (W.M.); pieter-jan.guns@uantwerpen.be (P.-J.G.)

**Keywords:** arterial stiffness, vascular reactivity, cardiac function, proteasome inhibitors, bortezomib, carfilzomib

## Abstract

Proteasome inhibitors such as bortezomib and carfilzomib induce apoptosis and are a cornerstone in the treatment of relapsed or refractory multiple myeloma. However, concerns have emerged concerning their link to cancer therapy-related cardiovascular dysfunction (CTRCD). Bortezomib, a reversible first-generation inhibitor, and carfilzomib, a second-generation irreversible inhibitor, are associated with hypertension, heart failure, and cardiac arrhythmias. The current study investigated the effects of bortezomib and carfilzomib on cardiac (left ventricular ejection fraction, LVEF) and vascular (arterial stiffness, vascular reactivity) function. Cardiac function assessment aimed to build upon existing evidence of proteasome inhibitors CTRCD, while arterial stiffness served as an early indicator of potential vascular remodeling. Groups of 12-week-old C57BL/6J male mice (*n* = 8 per group) were randomly assigned to receive vehicle, carfilzomib (8 mg/kg I.P.), or bortezomib (0.5 mg/kg I.P.). Additionally, proteasome inhibition was assessed in mice treated with L-NAME (0.5 mg/kg) to induce hypertension. Cardiac and vascular parameters were evaluated via echocardiography on days 0 and 3. On day 6, mice were sacrificed for ex vivo analysis of arterial stiffness and vascular reactivity. Overall, no changes in arterial stiffness were detected either in vivo or ex vivo at basal pressures. However, a steeper pressure–stiffness curve was observed for carfilzomib in normotensive (*p* < 0.01) and hypertensive (*p* < 0.0001) mice ex vivo. Additionally, in hypertensive mice, carfilzomib decreased LVEF (*p* = 0.06), with bortezomib exhibiting similar trends. Vascular reactivity remained largely unchanged, but proteasome inhibition tended to enhance endothelial-independent relaxations in both control and hypertensive mice. In conclusion, short-term treatment with carfilzomib and bortezomib is considered relatively safe for the protocols assessed in the study.

## Simple Summary

Proteasome inhibitors, such as bortezomib and carfilzomib, are vital for treating relapsed or refractory multiple myeloma, but their potential link to cancer therapy-related cardiovascular dysfunction (CTRCD) raises concerns. Bortezomib, a reversible first-generation inhibitor, and carfilzomib, a second-generation irreversible inhibitor, have been associated with hypertension, heart failure, and arrhythmias. This study explored their impact on cardiac function, focusing on left ventricular ejection fraction (LVEF), and vascular function through arterial stiffness and vascular reactivity assessments. Twelve-week-old male mice were treated with either carfilzomib or bortezomib, with some receiving L-NAME to induce hypertension. Echocardiography was used to evaluate cardiac and vascular parameters at different time points, followed by ex vivo arterial stiffness and reactivity measurements. Results showed no significant changes in arterial stiffness at baseline pressures, but a steeper pressure-stiffness curve was noted in carfilzomib-treated normotensive and hypertensive mice. Carfilzomib also showed a trend toward reduced LVEF in hypertensive mice, with bortezomib showing similar trends. Vascular reactivity remained largely unchanged, but proteasome inhibition appeared to enhance endothelial-independent relaxation. Overall, short-term treatment with both drugs was deemed relatively safe under the tested conditions.

## 1. Introduction

Proteasome inhibitors represent a crucial class of pharmacological agents used in multiple myeloma therapy (MM), renowned for their ability to disrupt the proteolytic pathway responsible for protein degradation [1]. Bortezomib and carfilzomib, prominent members of this class, exhibit significant efficacy in inducing apoptosis in cancer cells [2,3].

However, a growing body of evidence has raised concerns about a potential link between proteasome inhibition and cancer therapy-related cardiac dysfunction (CTRCD) [4]. Bortezomib, a reversible first-generation proteasome inhibitor widely used in MM therapy, has been associated with reports of hypertension, heart failure, and cardiac arrhythmias, prompting a re-evaluation of its impact on cardiovascular safety [5]. Similarly, carfilzomib, an irreversible second-generation proteasome inhibitor has also been linked with reports of cardiotoxicity including hypertension, heart failure, and cardiac arrhythmias, necessitating a nuanced understanding of proteasome inhibition in the context of CTRCD [6]. Both bortezomib and carfilzomib exert their therapeutic effects by disrupting the ubiquitin–proteasome pathway [1,3]. Bortezomib selectively binds to the 26S proteasome subunit and subsequently inhibits chymotrypsin-like activity. Carfilzomib displays enhanced specificity toward the chymotrypsin-like active site, resulting in the inhibition of both chymotrypsin-like and caspase-like activities. These mechanisms lead to the accumulation of proteins within cancer cells, ultimately triggering apoptosis [1,3].

In a recent study conducted in South Korean hospitals, Jang et al. investigated CTRCD associated with bortezomib in patients with MM, revealing that 20.8% of patients experienced CTRCD of any grade, with 14.7% experiencing severe adverse events [7]. These rates were notably higher than those reported in a meta-analysis (3.8%) [8] and phase 3 clinical trial (15%) [9]. A retrospective analysis of MM patients treated with carfilzomib reported cardiac and vascular-related adverse events, with patients showing symptom improvement after discontinuation of therapy [10]. Further evaluation of the Food and Drug Administration (FDA) Adverse Event Reporting System (FAERS) database (2014–2019) revealed significant associations of carfilzomib with CTRCD, including cardiomyopathy-related complications, embolic and thrombotic events, as well as cardiac failure [11]. The hypothesis was that these non-hematologic adverse events were related to the effects of proteasome inhibitors on the cardiovascular system, possibly mediated through changes in endothelial nitric oxide synthase (eNOS) activity and nitric oxide (NO) levels [10]. Endothelial dysfunction, linked with impaired vasodilation, was suggested as a potential mechanism contributing to cardiac dysfunction [11,12]. Furthermore, endothelial dysfunction is a known contributor to the development of arterial stiffness [10,12,13,14,15]. Therefore, arterial stiffness is suggested as an early marker for identifying adverse vascular outcomes. 

Carfilzomib is a known inducer of endothelial dysfunction [10]. In a prospective study, observations were made that carfilzomib therapy adversely affected endothelial function in patients with relapsed or refractory MM, as evidenced by impaired flow-mediated dilation (FMD), both acutely (first dose) and long-term (cycles 3 and 6) [13]. Interestingly, a recent study in mice demonstrated that carfilzomib-induced cardiotoxicity (decreased FS) was present following both a two and four-dose administration protocol [16,17]. Subsequent research investigating the vascular effects of proteasome inhibition found that carfilzomib induced vascular hypo-contraction and increased reactive oxygen species (ROS) while exhibiting no discernible impact on endothelial cell relaxations. Moreover, a tendency towards increased collagen thickness in the murine aorta was observed following two doses of carfilzomib [18]. However, subacute (four-dose) administration did not lead to permanent vascular dysfunction, suggesting a reversible impact on vascular function [18]. Overall, the increases in aortic collagen thickness and reduced VSMC function are critical factors in regulating vascular function and subsequent arterial stiffness, warranting further investigations.

Collectively these reports support the hypothesis that proteasomal function may negatively impact cardiovascular homeostasis [17,18,19]. Therefore, the current study aimed to build on previous reports of carfilzomib-induced cardiac dysfunction. Additionally, the influence of reversible and irreversible proteasome inhibitors on vascular function was evaluated, particularly focusing on ex vivo arterial stiffness and vascular reactivity [20].

## 2. Methods

### 2.1. Animal Experimentation

**Figure 1:**
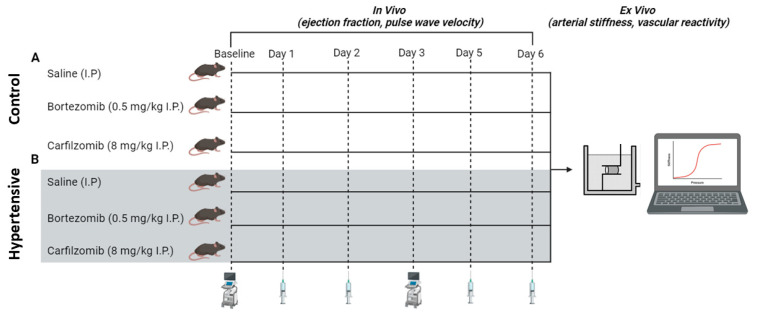
**Schematic representation of experimental protocols.** (**A**) Control mice cohort (*n* = 8 per group): evaluation of cardiac parameters by echocardiography was performed on day 0 (baseline) and day 3. At day 6, all mice were sacrificed for ex vivo analysis. I.P. injections were performed on days 1, 2, 5, and 6. (**B**) Hypertensive mice cohort (*n* = 8 per group): mice underwent pre-treatment with L-NAME (2 mg/mL, drinking water) for 7 days preceding the administration of proteasome inhibitors, and maintained throughout the experimental procedure. The experimental protocol was repeated exactly as seen with control mice (**A**). **I.P.** = intraperitoneal.

Twelve-week-old C57BL/6J male mice (*n* = 8 per group) were purchased from Charles River (Ecully, France). Mice were then subjected to experimental protocol A (normotensive “control” mice) or B (L-NAME induced hypertension; Figure 1). All mice were housed in the University of Antwerp’s animal facility in standard cages, maintained under a 12 h light/dark cycle. Mice had unrestricted access to regular chow and tap water. The housing environment was controlled at a constant room temperature of 20–24 °C and a humidity level of 45%. The animal procedures conformed to ARRIVE guidelines and Directive 2010/63/EU of the European Parliament on the protection of animals used for scientific purposes and all experiments were approved by the ethics committee of the University of Antwerp (File 2023-40). Given the common occurrence of cardiovascular comorbidities, such as hypertension, in cancer patients, our objective was to increase the translational significance of our findings by adding a hypertensive mouse model. To induce hypertension, a cohort of mice underwent pre-treatment with L-NAME (2 mg/mL, drinking water) for 7 days preceding the administration of proteasome inhibitors, and maintained throughout the experimental procedure. Mice were randomly assigned to the following groups: vehicle (saline intraperitoneally, I.P.), bortezomib (0.5 mg/kg I.P.), carfilzomib (8 mg/kg I.P.), L-NG-Nitro arginine methyl ester (L-NAME 2 mg/mL + saline I.P.), L-NAME + bortezomib (2 mg/mL + 0.5 mg/kg I.P.) or L-NAME + carfilzomib (2 mg/mL + 8 mg/kg I.P.). I.P. injections were performed on days 1, 2, 5 and 6. The current study’s focus was on the acute treatment effects. Drug dose, treatment duration and route of administration were established based on previous research by Efentakis et al. [17,18]. Therefore, our daily doses following conversion were 0.5 mg/kg bortezomib and 8 mg/kg carfilzomib. For protocols A and B, in vivo evaluation of cardiac parameters by echocardiography was performed on day 0 (baseline) and day 3. At day 6, all mice were sacrificed for ex vivo arterial stiffness and vascular tone analysis.

### 2.2. Ultrasound Imaging of Cardiovascular Function

Ultrasound imaging was performed under isoflurane (1.5–2.5% (*v*/*v*) (Forene; Abbvie, Belgium) anesthesia using a high-frequency ultrasound system (Vevo2100, VisualSonics). Data acquisition commenced only when the heart rate (550 ± 50 beats per minute) and body temperature (37 °C) met the inclusion criteria. Cardiac parameters were assessed via M-mode imaging using a 24-MHz transducer. Left ventricular ejection fraction (LVEF), FS, left ventricular internal diameter (LVID), left ventricular anterior wall (LVAW) and left ventricular posterior wall (LVPW) thickness were subsequently calculated using measurements of three consecutive M-mode cycles with Vevo LAB Software (Version 3.2.0, Fujifilm VisualSonics, Toronto, Canada). Pulse wave velocity (PWV) was measured in the abdominal aorta using a previously described method [21]. In brief, PWV was calculated by measuring the transit time of the pulse waveform at two sites along the vasculature through electrocardiogram-gated kilohertz visualization (EKV), thus providing a local assessment of aortic stiffness.

### 2.3. Blood Pressure Evaluation

Systolic blood pressure (BP), diastolic BP and mean BP were determined non-invasively in restrained, awake mice using a tail-cuff system with a programmed electro sphygmomanometer (Coda, Kent Scientific Corporation, Torrington, CT, USA). Mice were trained one day before the actual measurements to reduce stress and variability during measurements. To this end, the cuff system was placed on the mouse tail as performed during the actual measurements. The duration of the training and measurement sessions was 30 min. Measurements were only performed in the L-NAME cohort at baseline and post-third injection.

### 2.4. Ex Vivo Arterial Stiffness 

Ex vivo stiffness of aortic segments was determined via a Rodent Oscillatory Set-up to Study Arterial Compliance (ROTSAC) as previously described by Leloup et al. [22]. In brief, 2 mm thoracic aortic segments were mounted between two parallel hooks in 10 mL organ baths. Segments were immersed in Krebs Ringer (KR) solution (37 °C, 95% O2/5% CO2, pH 7.4) containing (in mM): NaCl 118, KCl 4.7, CaCl2 2.5, KH2PO4 1.2, MgSO4 1.2, NaHCO3 25, CaEDTA 0.025 and glucose 11.1. Force and displacement of the upper hooks were controlled and assessed using a force-length transducer. Segments were subjected to cyclic stretching, between alternating preloads, emulating “diastolic” and “systolic” transmural pressures at 10 Hz frequency mimicking the physiological heart rate in mice (600 bpm). The ROTSAC protocol for all experiments included the evaluation of arterial stiffness (Peterson pressure strain modules of elasticity (Ep)) at different pressures (i.e., ranging from 60–100 mmHg to 180–220 mmHg with 20 mmHg incremental intervals), under a physiological (Krebs–Ringer solution) condition.

### 2.5. Evaluation of Vascular Reactivity 

Thoracic aortic segments (2 mm) were configured to a constant preload of 20 mN equivalent to 100 mmHg mean pressure [23]. VSMC contraction was assessed by administering cumulative concentrations of phenylephrine (PE; 3 nM–3 µM), an α1-adrenergic receptor agonist. Endothelial-dependent relaxations were then evaluated using cumulative concentrations of acetylcholine (ACh; 3 nM–3 µM), a muscarinic receptor agonist. To eliminate the influence of NO, L-NAME (300 µM), a non-selective NO synthase inhibitor, was introduced. Additionally, 2-(N,N-diethylamino)-diazenolate-2-oxide sodium salt hydrate (DEANO; 0.3 nM–10 µM), an exogenous nitric oxide donor, was administered to assess VSMC sensitivity to NO independent of endothelial function. Additionally, 50 mM potassium was used to induce contractions independent of NO by way of membrane depolarization.

### 2.6. Cell Viability Assay Through Neutral Red Uptake

In vitro-induced cytotoxicity following 24 h incubation of bortezomib (3 nM, 30 nM, 300 nM, 3 mM) or carfilzomib (3 nM, 30 nM, 300 nM, 3 mM) was evaluated on murine vascular smooth muscle cells (mVSMC), human aortic smooth muscle cells (HaoSMC), as well as mesenchymal progenitor cardiac endothelial cells (MPCEC). Cytotoxicity is expressed as a concentration-dependent reduction in uptake of neutral red after exposure.

### 2.7. Chemical Compounds

Carfilzomib was purchased from Tebu-Bio (product I.D T1795). Bortezomib (product I.D 5043140001), PE, L-NAME, and DEANO were obtained from Sigma-Aldrich (Overijse, Belgium). Anti-Proteasome 20S alpha + beta antibody was purchased from Abcam (ab22673).

### 2.8. Statistics

All results are expressed as mean ± standard error of the mean (SEM) with n representing the number of mice. Statistical analyses were performed in GraphPad Prism 9.0. Statistical tests are mentioned in the figure legends. Significance was accepted at *p* < 0.05.

## 3. Results

Importantly, our in vitro findings revealed that the dose of carfilzomib and bortezomib employed in our study did not induce cell death (Supplementary Figure S1). Subsequently, the echocardiographic parameters of control and hypertensive mice treated with saline, carfilzomib (8 mg/kg), or bortezomib (0.5 mg/kg) are presented in Table 1. Following carfilzomib or bortezomib treatment, subtle changes in in vivo echocardiographic parameters were observed. Particularly, FS, an indicator of left ventricular (LV) function, demonstrated attenuation (*p* < 0.05) in hypertensive mice after carfilzomib treatment, while a trend was observed for bortezomib.

**Figure 2:**
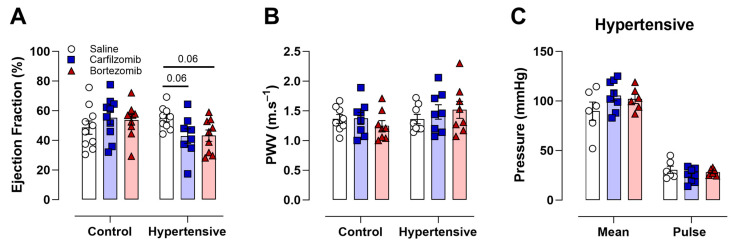
**Cardiac function and blood pressure following carfilzomib or bortezomib treatment.** (**A**) Ejection fraction was unaltered following either carfilzomib (8 mg/kg) or bortezomib (0.5 mg/kg) treatment in control mice. In hypertensive mice, ejection fraction tended to be lower after carfilzomib (8 mg/kg) or bortezomib (0.5 mg/kg) treatment. (**B**) PWV was unaltered following proteasome treatment in both control and hypertensive mice. (**C**) No differences were observed in mean blood pressure and pulse pressure values in hypertensive mice following saline, carfilzomib (8 mg/kg), bortezomib (0.5 mg/kg), and treatment. Statistical analyses: (**A**,**B**) one-way ANOVA with Sidak post hoc test for multiple comparisons, (**B**) two-way ANOVA with Sidak post hoc test for multiple comparisons. *n* = 8 per group.

Notably, no differences were observed in LVEF in treated control mice (Figure 2A). Conversely, in hypertensive mice, both carfilzomib (*p* = 0.06) and bortezomib (*p* = 0.06) treatments showed a trend for a reduced LVEF (Figure 2A). This suggests that the administration of carfilzomib (8 mg/kg) or bortezomib (0.5 mg/kg) can potentially induce systolic cardiac dysfunction in hypertensive mice. The administration of either carfilzomib or bortezomib to control and hypertensive mice did not lead to changes in in vivo arterial stiffness, as assessed using PWV (Figure 2B). Mean blood pressure and pulse pressure, surrogate markers of arterial stiffness, did not show changes in hypertensive mice following carfilzomib or bortezomib treatment (Figure 2C), while the L-NAME treated groups (i.e., hypertension cohorts) showed increased blood pressure.

The general characteristics of control and hypertensive mice treated with saline, carfilzomib (8 mg/kg), or bortezomib (0.5 mg/kg) are presented in Table 2. Carfilzomib administration in control mice resulted in a reduction in body weight (*p* < 0.001), with a trend toward lower body weights observed for bortezomib. However, no differences were observed in heart weight/body weight ratio following carfilzomib and bortezomib treatment in control mice. Interestingly, in hypertensive mice, neither body weight nor heart weight/body weight ratio was altered following carfilzomib or bortezomib treatment.

**Figure 3:**
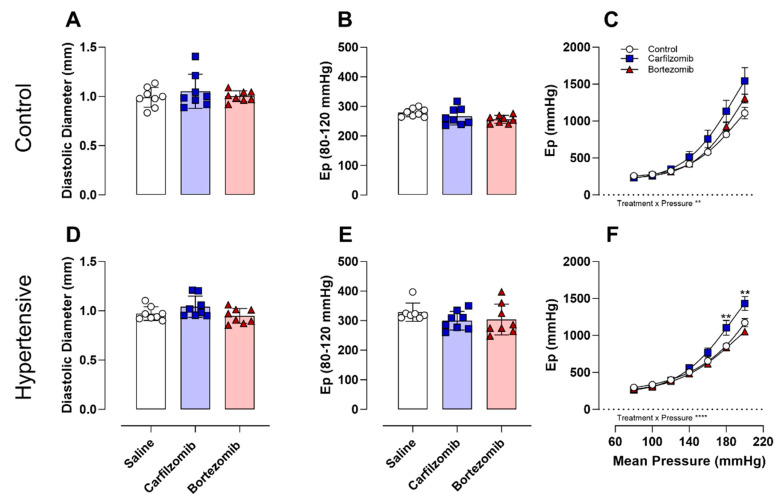
**Pressure–stiffness response to proteasome inhibition.** Diastolic diameters were unaltered at basal pressures (**A**,**D**). No differences were reported in arterial stiffness (Ep) under basal pressures (**B**,**E**). Observed differences in pressure–stiffness curve morphology were evident in control and hypertensive mice under physiological conditions (**C**,**F**). Statistical analyses: one-way ANOVA with Sidak post hoc test for multiple comparisons (A, B, D, E). Two-way ANOVA with Sidak post hoc test for multiple comparisons (C, F). *n* = 8 per group, ** *p* < 0.01, **** *p* < 0.0001. **Ep** = Peterson’s elastic modulus, **DEANO** = 2-(N, N-diethylamino)-diazenolate-2-oxide sodium salt hydrate.

After in vivo evaluations, arterial stiffness was examined ex vivo following treatment with carfilzomib and bortezomib under physiological conditions in both control and hypertensive mice (Figure 3A–F). In control mice, no differences in diastolic diameters (Figure 3A) or arterial stiffness (Figure 3B) were reported at basal (i.e., 80–120 mmHg) pressure; however, a treatment interaction effect (*p* < 0.01) was noted in the pressure–stiffness relationship (Figure 3C). Similarly, evaluation in hypertensive mice revealed no differences in diastolic diameters (Figure 3D) or arterial stiffness (Figure 3E) at basal (i.e., 80–120 mmHg) pressure. Yet, treatment interaction effects (*p* < 0.0001) and treatment effects (*p* < 0.01) in the pressure–stiffness relationship (Figure 3F) were reported. Notably, carfilzomib experienced elevated (*p* < 0.01) arterial stiffness at the highest mean pressures (i.e., 180, 200 mmHg). Elevated basal stiffness values (80–120 mmHg) observed in saline-treated hypertensive mice compared to saline-treated control mice served as internal validation of our model (Ep controls: 279 ± 14 vs. Ep hypertensive: 329 ± 12 (*p* < 0.01)).

**Figure 4:**
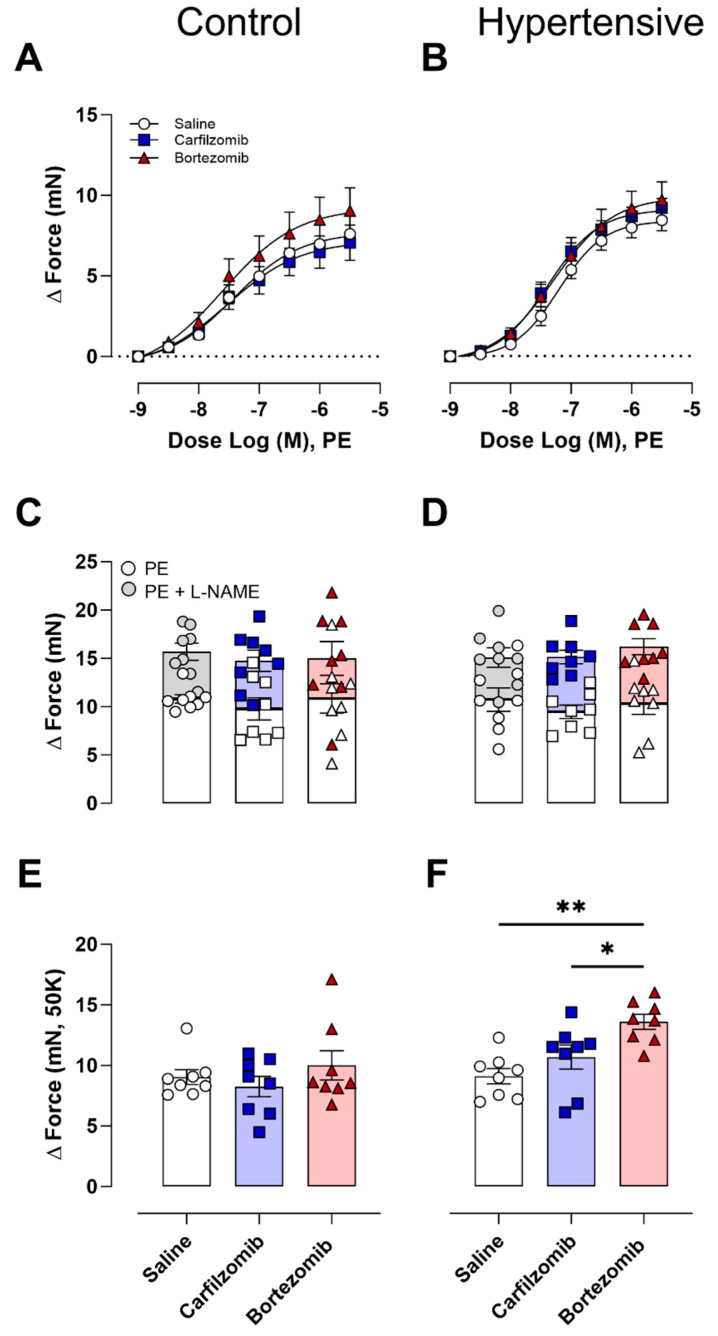
**Vasoconstriction following proteasome inhibition.** Receptor-mediated contractions assessed with PE revealed no differences following bortezomib and carfilzomib treatment in control (**A**) and hypertensive (**B**) mice. Contraction in the presence (PE) and absence (PE+ L-NAME) of NO (**C**,**D**) revealed no treatment differences. Additionally, potassium contractions revealed no changes observed in control mice (**E**), while treatment differences were reported in hypertensive mice (**H**). Statistical analyses: two-way ANOVA with Sidak post hoc test for multiple comparisons (**A**,**B**). One-way ANOVA with Sidak post hoc test for multiple comparisons (**C**–**F**). *n* = 8 per group, * *p* < 0.05; ** *p* < 0.01. PE = phenylephrine, LN = L-NG-nitro arginine methyl ester, 50 K = 50 mM potassium.

Following the ex vivo assessment of arterial stiffness, the impact of carfilzomib and bortezomib on vascular reactivity in the control and hypertensive mice was investigated. Neither carfilzomib nor bortezomib affected VSMC contractions induced by PE (Figure 4A,B). Additionally, maximal VSMC contractions in the presence and absence (Figure 4C,D) of NO did not demonstrate any treatment effects from carfilzomib or bortezomib. Moreover, NO-independent contractions stimulated by potassium (50 mM) in control mice revealed no treatment differences (Figure 4E). However, hypertensive mice demonstrated treatment disparities, with bortezomib resulting in heightened contractility (bortezomib vs. control, *p* < 0.01; bortezomib vs. carfilzomib, *p* < 0.05) (Figure 4F).

**Figure 5:**
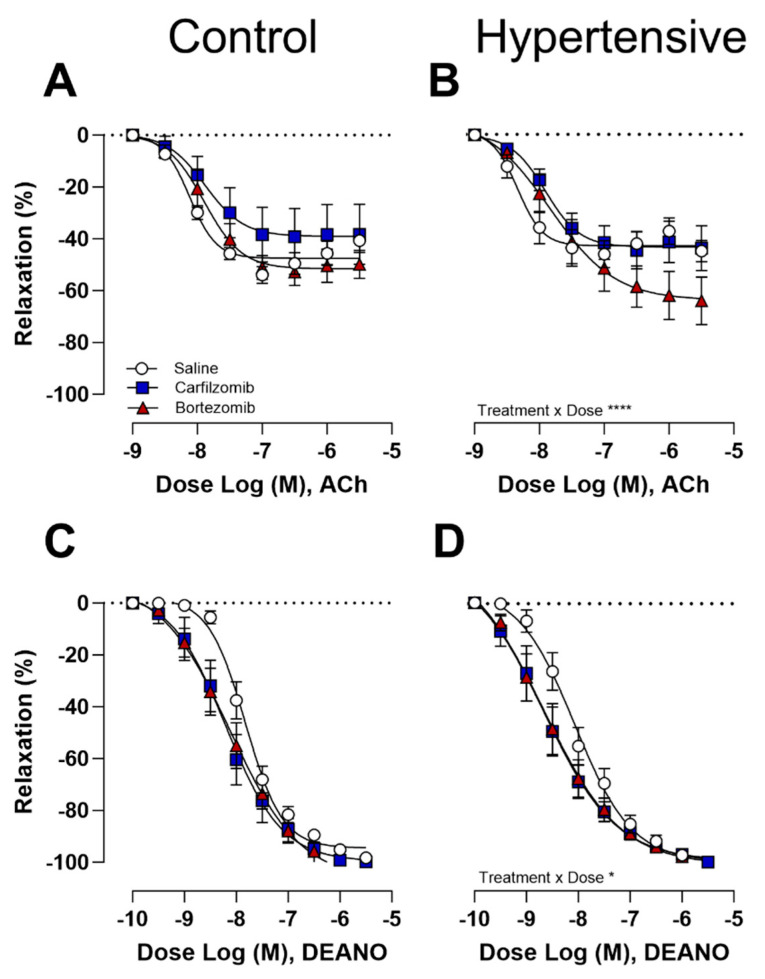
**Vasodilation following proteasome inhibition.** Endothelium-dependent vasorelaxation with acetylcholine revealed no treatment effects in control mice (**A**). Yet, a heightened relaxation was observed under bortezomib in hypertensive (**B**) mice. No differences were reported following endothelium-independent vasorelaxation with DEANO; however, a trend was present for both control (**C**) and hypertensive (**D**) mice. Two-way ANOVA with Sidak post hoc test for multiple comparisons. *n* = 8 per group, * *p* < 0.05; **** *p* < 0.0001. **ACh** = acetylcholine, **DEANO** = 2-(N,N-diethylamino)-diazenolate-2-oxide sodium salt hydrate.

Endothelial-dependent relaxations in response to ACh did not exhibit differences following carfilzomib or bortezomib treatment in control mice (Figure 5A). However, in hypertensive mice, bortezomib reported a trend towards heightened relaxations (Figure 5B), with an interaction effect between treatments present (*p* < 0.0001). Interestingly, a left-shift in endothelial-independent relaxations was evident under DEANO, indicating a trend towards increased sensitivity to the NO donor in both control (Figure 5C) and hypertensive mice (Figure 5D), although statistically non-significant.

## 4. Discussion

The current study employed a comprehensive approach encompassing in vivo and ex vivo assessments to investigate the cardiovascular impact of short-term carfilzomib and bortezomib therapy. Given that cancer patients frequently present with cardiovascular comorbidities such as hypertension, an L-NAME-induced hypertensive mouse cohort was added to increase the translational significance of our study. Short-term proteasome inhibition resulted in limited effects with only hypertensive mice reporting a trend towards a drop in systolic left ventricle function. For carfilzomib, we observed increased ex vivo stiffness at the higher end of the pressure–stiffness curves across both experimental models, and more pronounced in hypertensive mice, while no changes in PWV were observed in vivo. We attribute this observation to numerically higher basal diameters in the carfilzomib-treated mice leading to earlier recruitment of collagen fibers and hence an increased pressure–stiffness profile. Overall, ex vivo vascular reactivity remained largely unchanged, although, a trend was present where proteasome inhibition enhanced the sensitivity of VSMCs to exogeneous NO across both experimental models.

### 4.1. In Vivo Cardiovascular Assessment

Carfilzomib and bortezomib have previously been shown to induce cardiotoxicity, evidenced by decreased FS and LVEF, in animal models as well as patients with MM [4,16,24,25,26]. Carfilzomib has been linked to an increased occurrence of CTRCD compared to bortezomib, partly attributed to its irreversible proteasome inhibition [4,27]. Drug dose, treatment duration and the route of administration were established based on previous research by Efentakis et al. [17,18]. Consequently, the four-dose administration protocol aligns with an approach mimicking clinical treatment schedules demonstrated in patients undergoing MM therapy. In humans, the European medicine agency advises a single dose of 1.4 mg/m^2^ bortezomib [25] and 24 mg/m^2^ carfilzomib [26] per day for treating MM. A Km division factor of 0.3 was implemented as previously suggested [28]. Importantly, our in vitro findings revealed that the dose of carfilzomib and bortezomib employed in our study did not induce cell death, indicating these compounds were not cytotoxic at the tested doses. Notably, in our current study, the administration of carfilzomib and bortezomib in control mice did not lead to impaired cardiac function, while hypertensive mice showed slight deterioration in cardiac parameters, particularly manifesting as reduced LVEF and FS. Patients with MM commonly manifest pre-existing cardiovascular conditions, including hypertension [27,29,30]. Hypertensive mice, comparable to the above-mentioned clinical scenarios, already bear an elevated cardiac afterload due to heightened blood pressure [31]. Consequently, drug treatment effects may be more pronounced in this context, exacerbating hypertensive conditions and further impairing the heart’s ability to effectively pump blood [32]. Our current study shows partial disagreement with the findings reported by Efentakis et al. [16]. However, in our study, echocardiography was conducted following two doses as the potential influence of anesthesia on vasoactive signaling pathways, vascular cell reactivity, and vascular tone, as documented in previous studies [33,34,35], rendered the in vivo cardiac assessment after four doses impractical. Therefore, our in vivo results should be compared to those of the two-injection protocol by Efentakis et al. [16]. Importantly, Efentakis et al. reported a more pronounced cardiotoxicity and cardiomyopathy present in mice after a four-injection protocol compared to a two-injection protocol, as represented by a decline in FS [16]. Thus, considering these observations, it is conceivable that cardiotoxicity could manifest in both murine models. Furthermore, no alterations in arterial stiffness, as measured by PWV, were documented in either control or hypertensive mice treated with carfilzomib and bortezomib, suggesting the absence of any discernible in vivo vascular treatment effects, although pulse pressure and mean blood pressure tended to be increased following treatment. Additionally, our study did not observe the anticipated increases in PWV measurements following L-NAME treatment as previously reported [36]. Interestingly, a decrease in heart weight was observed in control mice, an effect not replicated in hypertensive mice. Nevertheless, we attribute this observation to an artefact rather than a biological effect, given the minimal absolute changes observed and comparable heart weights to body weight ratios. However, a potential explanation may be that baseline heart weights in hypertensive mice are elevated, obscuring any treatment-related decreases observed in hypertensive mice [27,37].

### 4.2. Insights into Arterial Stiffness and Vascular Reactivity

Ex vivo analyses of arterial stiffness enabled the evaluation of vascular function independently from in vivo confounding factors (i.e., heart rate and blood pressure). In alignment with our in vivo assessments, no clear differences in arterial stiffness were detected under basal pressure conditions (80–120 mmHg). However, treatment with carfilzomib resulted in slightly higher numerical diameter values within this pressure range. Moreover, a steeper pressure–stiffness relationship was observed for carfilzomib, suggesting the pressure-dependent increases in arterial stiffness may be attributable to geometrical remodeling, potentially involving the recruitment of stiffer collagen fibers at lower pressures [38]. Previously, carfilzomib therapy in patients with MM has been associated with adverse endothelial function, evident by impaired flow-mediated dilation and diminished NO bioavailability [10,12,13,14,15]. Similarly, in mice, carfilzomib was shown to worsen endothelial cell-mediated vasorelaxation, measured by ex vivo aortic tension assessment [18]. However, this was not reproduced in our current study where endothelial-dependent relaxations showed no obvious deterioration following treatment. Moreover, despite the administration of L-NAME, an eNOS blocker, to the hypertensive cohort, no distinct indications of endothelial dysfunction were reported ex vivo, potentially attributed to a washout effect or resynthesis of eNOS, and in line with a previous study by our laboratory [36]. Interestingly, heightened sensitivity to DEANO, demonstrated by leftward shifts in relaxation curves, was consistently observed in both control and hypertensive mice following carfilzomib and bortezomib treatment. Proteasome inhibitors, known inducers of oxidative stress, potentially alter protein turnover in the soluble guanylate cyclase (sGC) pathway within VSMCs, leading to reduced sGC levels and possibly sensitizing receptors to exogenous NO [39,40]. However, contractions in the presence or absence of L-NAME did not reveal any treatment differences, aligning with prior research conducted by Efentakis et al., where subacute (four-dose) carfilzomib treatment did not influence vascular contractility, while acute (two-dose) administration resulted in reduced vascular contractility, thereby adding to the conflicting data concerning the vascular consequences of proteasome inhibition [18]. Potassium-induced contractions were unchanged in the aorta segments of the control mice but increased in the hypertensive mice treated with bortezomib. From a physiological/pharmacological perspective, we do not see a clear explanation for the difference in 50 mM K^+^-induced contractions by bortezomib versus the PE-induced contraction in the presence of L-NAME. A possible, yet rather speculative explanation might be that proteasome inhibition impacts ion channel protein turnover and calcium regulation in VSMCs [41,42].

### 4.3. Limitations

A clear limitation of the current study is the relatively short exposure time and lack of longer-term follow-up, as extended observation periods could provide valuable insights into the sustained effects of proteasome inhibition on cardiovascular dysfunction. Additionally, ex vivo measurements employed in the study may not fully replicate the acute in vivo effects of drugs due to the potential wash-out effect. However, increased basal arterial stiffness values observed ex vivo in L-NAME-treated mice served as internal model validation. Furthermore, the intraperitoneal administration of carfilzomib and bortezomib does not reflect the clinical protocols that employ intravenous (carfilzomib) or intravenous/subcutaneous (bortezomib) routes. Although prior research, including studies by Efentakis et al., (17, 18) has shown that intraperitoneal administration in mouse models can achieve proteasome inhibition concentrations comparable to those observed in clinical settings, it is important to recognize that intravenous administration may show different pharmacokinetics, potentially explaining the lack of effect noted in this current study. Finally, multiple myeloma patients are often elderly patients (>70 years) [43]. In retrospect, it would have been interesting to evaluate proteasome inhibition in aged mice. Aged mice have been described as displaying endothelial dysfunction and as such may be more susceptible to proteasome inhibition. On the other hand, we did evaluate proteasome inhibition in an experimental model of hypertension, a cardiovascular comorbidity, the prevalence of which increases with ageing. Nevertheless, future investigations may better use old(er) mice to align with the clinical characteristics of the patient population.

## 5. Conclusions

In conclusion, our results showed that treatment with carfilzomib and bortezomib resulted in limited cardiac and vascular effects over the short-term (four-injection) protocols assessed, suggesting their safety under short-term evaluation protocols. However, further research is needed to assess the long-term effects of reversible and irreversible proteasome inhibition on arterial stiffness and vascular toxicity, as well as across different models of cardiovascular disease. Additionally, the inclusion of ixazomib, another second-generation FDA-approved orally administered proteasome inhibitor, could provide valuable insights [44].

## Figures and Tables

**Table 1:** Echocardiographic parameters of control and hypertensive male (C57BL6/J) mice treated with saline, carfilzomib or bortezomib.

Group	Control			Hypertensive		
Treatment	Saline	Carfilzomib	Bortezomib	Saline	Carfilzomib	Bortezomib
	Mean	Mean	Mean	Mean	Mean	Mean
**LVAW; d** (mm)	0.8 ± 0.1	0.9 ± 0.1	0.8 ± 0.1	0.8 ± 0.1	0.8 ± 0.1	0.9 ± 0.1
**LVAW; s** (mm)	1.1 ± 0.1	1.2 ± 0.1	1.1 ± 0.1	1.1 ± 0.1	1.1 ± 0.1	1.1 ± 0.1
**LVID; d** (mm)	3.9 ± 0.2	3.6 ± 0.1	3.8 ± 0.1	3.7 ± 0.2	4.1 ± 0.2	3.9 ± 0.2
**LVID; s** (mm)	3.0 ± 0.2	2.5 ± 0.2	2.8 ± 0.1	2.7 ± 0.2	3.2 ± 0.2	3.1 ± 0.2
**LVPW; d** (mm)	0.9 ± 0.1	0.8 ± 0.1	0.8 ± 0.1	0.9 ± 0.1	0.9 ± 0.1	0.9 ± 0.1
**LVPW; s** (mm)	1.1 ± 0.1	1.2 ± 0.1	1.1 ± 0.1	1.2 ± 0.1	1.1 ± 0.1	1.2 ± 0.1
**FS** (%)	23.2 ± 2.6	30.9 ± 2.5	27.7 ± 2.2	28.2 ± 1.7	20.2 ± 2.2 *	22.1 ± 2.5
**LV Mass AW** (mg)	122.2 ± 12.8	101.9 ± 5.7	110.7 ± 6.0	116.2 ± 7.7	126.3 ± 14.6	148.1 ± 21.2
**LV Vol; d** (uL)	67.2 ± 6.4	55.5 ± 4.6	62.9 ± 3.0	58.4 ± 5.8	71.5 ± 7.1	70.6 ± 6.7
**LV Vol; s** (uL)	37.6 ± 5.1	23.9 ± 3.9	29.3 ± 2.9	27.1 ± 3.9	42.7 ± 6.2	40.4 ± 6.4

Data are represented as mean ± SEM. Statistical analysis using a one-way ANOVA with Sidak’s post hoc test for multiple comparisons (comparisons were made between the saline, bortezomib and carfilzomib group of either control of hypertensive mice for each parameter). * *p* < 0.05. LVAW; d = left ventricular anterior wall thickness in diastole; LVAW; s = left ventricular anterior wall thickness in systole; LVID; d = left ventricular internal diameter in diastole; LVID; s = left ventricular internal diameter in systole; LVPW; d = left ventricular posterior wall thickness in diastole; LVPW; s = left ventricular posterior wall thickness in systole; FS (%) = fractional shortening; LV Mass AW = left ventricular mass of anterior wall; LV Mass AW (corrected) = corrected left ventricular mass of anterior wall; LV Vol; d = left ventricular volume in diastole; LV Vol; s = left ventricular volume in systole. *n* = 8 per group.

**Table 2:** General characteristics of control and hypertensive male (C57BL6/J) mice treated with saline, carfilzomib or bortezomib.

Group	Control			Hypertensive		
Treatment	Saline	Carfilzomib	Bortezomib	Saline	Carfilzomib	Bortezomib
	Mean	Mean	Mean	Mean	Mean	Mean
**Body Weight** (g)	27.4 ± 0.7	23.5 ± 0.7 **	25.3 ± 0.5	27.6 ± 0.5	27.4 ± 0.7	27.9 ± 0.5
Δ **Body Weight** (g)	0.4 ± 0.4	−2.5 ± 0.5 ***	−0.9 ± 0.5	0.6 ± 0.4	0.4 ± 0.2	−0.1 ± 0.4
**Heart Weight** (mg)	140 ± 5	120 ± 4 *	116 ± 4 **	141 ± 5	148 ± 5	145 ± 4
**HW:BW** (1000×, g/g)	5.1 ± 0.2	4.9 ± 0.3	4.7 ± 0.1	5.1 ± 0.2	5.4 ± 0.2	5.2 ± 0.2

Data are represented as mean ± SEM. Statistical analysis using a two-way ANOVA with Sidak’s post hoc test for multiple comparisons, (comparisons were made between the saline, bortezomib and carfilzomib group of either control or hypertensive mice). Overall significance is shown in the final column. * *p* < 0.05, ** *p* < 0.01, *** *p* < 0.001; HW:BW = heart weight/body weight ratio. *n* = 8 per group.

## Data Availability

The datasets used and/or analyzed during the current study are available from the corresponding author (Callan Wesley) on reasonable request.

## Supplementary Materials

The following supporting information can be downloaded at https://www.mdpi.com/article/10.3390/biology13100844/s1, Figure S1: Cytotoxicity following bortezomib and carfilzomib incubation.
