# Supplementary material for: Short-Term Proteasome Inhibition: Assessment of the Effects of Carfilzomib and Bortezomib on Cardiac Function, Arterial Stiffness, and Vascular Reactivity
Source: Biology (Basel). 2024 Oct 21;13(10):844. doi: 10.3390/biology13100844 (PMC11504385; doi:10.3390/biology13100844)
Supplement: Supplementary file 1 [file biology-13-00844-s001.zip › biology-3230022-supplementary.pdf]

Supplementary Material

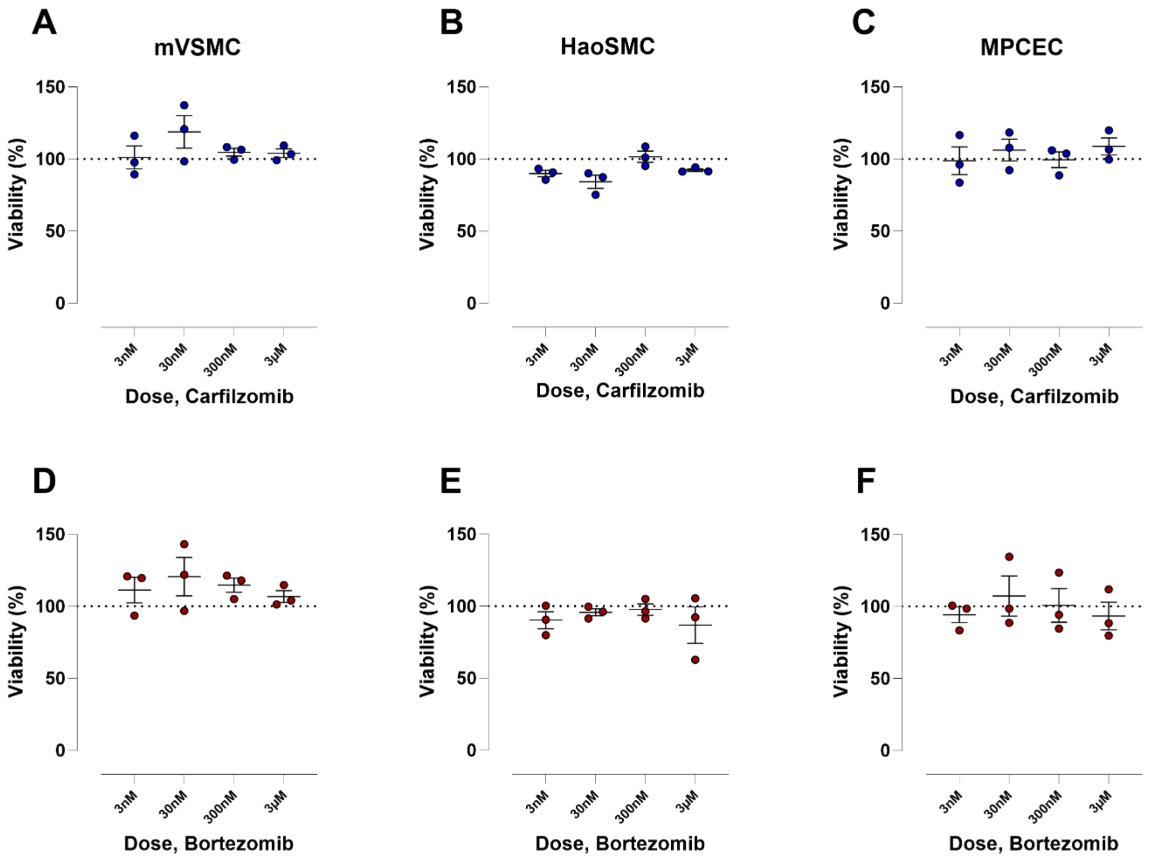

Supplementary Figure S1. Cytotoxicity following bortezomib and carfilzomib incubation. A 24-hour concentration response of bortezomib resulted in unalter cell viability in (A) mVSMC , (B) HaoSMC and (C) MPCEC. Additionally, (D) mVSMC, (E) HaoSMC and (F) MPCEC reported no decreased viability under carfilzomib treatment. mVSMC = murine vascular smooth muscle cells, HaoSMC = human aortic smooth muscle cells, MPCEC = mesenchymal progenitor endothelial cells. Statistical analyses: Two-way ANOVA with Sidak post-hoc test for multiple comparisons. n= 3.
